# Factors associated with common mental health problems of humanitarian workers in South Sudan

**DOI:** 10.1371/journal.pone.0205333

**Published:** 2018-10-31

**Authors:** Hannah Strohmeier, Willem F. Scholte, Alastair Ager

**Affiliations:** 1 Institute for Global Health and Development, Queen Margaret University, Edinburgh, Scotland; 2 Department of Anthropology, Yale University, New Haven, Connecticut, United States of America; 3 Antares Foundation, Amsterdam, Netherlands; 4 Amsterdam UMC, Department of Psychiatry, University of Amsterdam, Amsterdam, Netherlands; 5 Mailman School of Public Health, Columbia University, New York, New York, United States of America; CentERdata, NETHERLANDS

## Abstract

**Background:**

The latest data on major attacks against civilian aid operations have identified South Sudan as the most dangerous country for aid workers globally. Exposure to other traumatic events and chronic stress is also common in this population. No research exists on the mental health of humanitarian workers in South Sudan.

**Objectives:**

This study examined symptom burden and predictors of posttraumatic stress disorder (PTSD), depression, anxiety, hazardous alcohol consumption, and burnout among humanitarian workers in South Sudan.

**Method:**

We conducted a cross-sectional online survey with humanitarian workers (national and international staff, consultants, United Nations volunteers). We applied validated measures useful for this setting. We applied Least Absolute Shrinkage and Selection Operator (LASSO) regression to fit models with high prediction accuracy for each outcome and used ordinary least squares (OLS) regression to obtain final coefficients and perform inference.

**Results:**

A total of 277 humanitarian workers employed by 45 organizations completed the survey (a response rate in the order of 10%). We estimated prevalence of PTSD (24%), depression (39%), anxiety disorder (38%), hazardous alcohol consumption in men (35%) and women (36%), and the burnout components emotional exhaustion (24%) and depersonalization (19%). Chronic stress exposure was positively associated with PTSD (p < .001), depression (p < .001), anxiety (p < .001), emotional exhaustion (p < .01), and depersonalization (p < .001). We found no significant association between emotion focused and problem focused coping and mental health outcomes. Associations between dysfunctional coping and depression (p < .001) and anxiety (p < .01) were positive. Higher levels of spirituality were associated with lower risk of hazardous alcohol consumption (p < .001). Contrary to expectations, working directly with humanitarian aid beneficiaries was significantly associated with lower risk for emotional exhaustion (p < .01).

**Conclusion:**

Our results suggest that humanitarian workers in South Sudan experience substantial levels of mental ill-health. This study points to the need for staff support strategies that effectively mitigate humanitarian workers’ chronic stress exposure. The dynamics between coping and mental health among humanitarian workers require further study.

## Introduction

The humanitarian needs in countries affected by crises are at an unprecedented level [1], and South Sudan is one of the countries in which the situation is worse than ever [2]. The ongoing conflict and inter-communal violence accompanied by economic decline and climatic shocks in the world’s youngest nation have severe implications for its population [3]. The severity of the situation also bears a high level of risk for humanitarian workers operating in the country–dedicated professionals with the objective to alleviate the effects of the ongoing crisis. According to latest data from Humanitarian Outcomes [4,5], there were over thirty major attacks against civilian aid operations in 2015, and this increased to over fifty in 2016. This made South Sudan the most dangerous country for aid workers globally in both years [6,7]. The outbreak of violence in July 2016 in the country’s capital Juba exemplifies the magnitude of the situation. Among other atrocities, humanitarian workers have been gang-raped, injured and killed, and embassies and international organizations felt compelled to temporarily evacuate large parts of their civilian personnel [8,9]. The number of deaths of humanitarian workers since independence has just reached 100 [10].

In addition to physical harm, such exposure to conflict and violence, traumatic events and chronic stress can lead to post-traumatic stress disorder (PTSD). Depression and anxiety are among the most common comorbid conditions of PTSD and associated with war-related trauma and daily stressors [11,12]. Hazardous alcohol consumption is another common comorbid condition of PTSD [11,13]. Chronic stress at the workplace leads also frequently to burnout [14].

Some humanitarian workers manage life amidst crisis settings without developing symptoms of mental illness; some even seem to thrive in this type of work [15]. However, as previous studies confirm, PTSD, depression, anxiety, and burnout are indeed widespread among this occupation group; established prevalence rates are mostly similar to or higher than those of reference groups cited in the respective literature (e.g., [16–22]). Hazardous alcohol consumption received very little attention in the literature on humanitarian workers [23].

Regarding predictors, multiple studies confirm the positive association between chronic stress and traumatic event exposure and common mental health problems among humanitarian workers (e.g., [17,18,22,24,25]). Similarly, research identifies social support and team cohesion as important resilience factors among this occupation group (e.g., [17,18,20,22]). However, a number of gaps remain in the literature: Several studies analyzed the effect of organizational support, understood as the provision of benefits and services (e.g., medical insurance, counseling), or satisfaction with organizational culture (e.g., encouragement to take vacations). Lopes Cardozo et al. [22,26] and Eriksson et al. [27] found that both national and international staff satisfied with their organizations’ culture experience higher risk of mental ill-health. In contrast, neither the provision of benefits and services, nor the satisfaction with organizational culture was significantly associated with the mental health of national staff in the research undertaken by Ager et al. [17]. Some studies examined coping strategies, again with diverging results: Eriksson et al. [19] found avoidance coping to be positively associated with depression, anxiety, and PTSD in their study on pre-deployment mental health and trauma exposure of expatriate humanitarian workers. The results from Lopes Cardozo et al. [22] do not support the theory that avoidant coping presents a risk factor for PTSD among national staff. Few studies analysed the potential effects of healthy lifestyle habits (e.g., healthy eating, caffeeine intake, exercising) on humanitarian workers’ mental health [19,26]. While neither of these studies found a significant association between this predictor and mental health outcomes, the wider literature on humanitarian workers emphasizes the importance of healthy lifestyle habits in this context (e.g., [28,29]). Another construct that has gained recent attention in relation to coping is spiritual transcendence (e.g., [30,31]). However, quantitative research on its relation with the mental health of humanitarian workers has to our knowledge not yet been undertaken.

If mental health problems manifest, they come with serious implications for the individual’s personal and professional life [32]. Humanitarian workers themselves have increasingly spoken out on problems and voiced their urgent need for help (e.g. through public forums such as the Facebook group “Fifty Shades of Aid” and the “Secret Aid Worker” series published by The Guardian (e.g., [33,34]). Humanitarian organizations have taken note of the problem, and increasingly understand compromised mental health of their workforce as an issue that impacts negatively on organizational functioning; they have the obligation to prevent and address mental health problems among their staff, ensure their well-being and sustain organizational effectiveness and efficiency [35,36]. While these are desirable developments, major barriers in establishing new and adjusting existing staff support services and policies based on needs are the limited availability of evidence-based data and diverse findings on factors shaping the mental health of humanitarian workers: crisis settings differ greatly and staff support is most effective when tailored to the specific context [29]. Staff support is largely insufficient and underfunded and awareness building among donors is needed to ensure appropriate allocation of funds [37]. This is also acutely the case for South Sudan where humanitarian workers face a unique crisis [38], staff support is in need of improvement [39], and, to the best of our knowledge, no scientific research on the mental health of humanitarian workers (national and international staff, consultants, United Nations [UN] Volunteers) has previously been undertaken.

The distinction between national and international staff is commonly made in the humanitarian field on the basis of differences in contract modalities (e.g. access to security support), the nature of work undertaken (e.g. deployment in high-risk contexts), and the level of dependency on jobs in the sector to sustain livelihoods [40]. This has resulted in the majority of research in this field to date focusing on either national or international staff [16,21]. However, national and international staff also have many commonalities in their experience. The motives of both groups have been shown to reflect a complex combination of sense of challenge, humanitarian conviction, and personal interest, such as career advancement [41]. Issues of family adjustment and separation are reported by both national and international staff [42]. Clearly, humanitarian operations depend on the deployment of both within and integrated workforces. With differential treatment and expectation a noted source of resentment [43], this study applied an inclusive approach and explicitly sought to engage the humanitarian community in South Sudan as a whole. Further, our analysis did not presume being contracted as national or international staff to represent a basis for a priori separation of the sample into two distinct populations. Rather, as with a recent UNHCR study [44] on staff well-being, we saw this as one of a number of variables to be included in statistical analysis.

## Study objectives and hypotheses

Our study addresses the gap in research on the mental health of humanitarian workers in South Sudan through analyses of cross-sectional online survey data. Specifically, we aimed at establishing the symptom burden of five mental health outcomes–PTSD, depression, anxiety, hazardous alcohol consumption, and burnout–among this occupation group. We also aimed at fitting models with high predictive accuracy to assess relationships between predictor variables and mental health outcomes.

The Job Demands-Resources (JDR) model is a comprehensive and highly flexible model that is widely used to assess employee health, especially in the context of cross-sectional research [45,46]. It assumes that employee health results from a balance of negative job characteristics (demands) and positive job and personal characteristics (resources) [46]. Based on the JDR, job demands included in our models should be significantly positively related with the mental health outcomes under examination. Job and personal resources should be significantly negatively related with these.

## Methods

### Procedure and participants

One hundred forty-five humanitarian organizations were identified based on their participation in the 2017 South Sudan Humanitarian Response Plan and/or membership in the 2017 Humanitarian Country Team. Based on the availability of contact details, 124 organizations were contacted by email in April 2017 and requested to support an online survey. This support entailed designating a focal point within the organization who liaised with the researchers and disseminated the survey link, relevant information and two reminder emails to the workforce. To be included in the survey, organizations were required to have 10 or more staff employed in South Sudan and to have operated in the country for one year or longer. Those eligible for survey participation were national and international staff, consultants and UN Volunteers from supporting organizations whose official duty station was located in South Sudan.

The online survey was constructed in English (the working language in South Sudan), pilot tested on humanitarian workers with work experience in South Sudan and/or other major humanitarian crises, and adjusted based on the feedback received. The survey took approximately 45 minutes to complete, was launched in May 2017 and open for completion for one month.

### Ethical considerations

The study protocol was reviewed and approved by the Research Ethics Committee of Queen Margaret University, Edinburgh. Approval was granted on the basis of employing organizations being required to grant written permission following review of study goals and methods as outlined in the study information sheet. Informed consent was obtained from all survey participants. Given that the study involved surveying employees (rather than patients) and no personal identifying information, and given the evidence of the lack of a currently functioning national research ethics committee [47], this was considered the most appropriate means of securing necessary local approval consistent with country regulations.

### Measures

#### Mental health outcomes

We measured five common mental health outcomes: PTSD, depression, anxiety, hazardous alcohol consumption, and burnout. The validity and reliablity of the tools used to measure these outcomes were established in many countries and occupation groups (e.g., [48–51]).

PTSD symptoms: We used the PTSD Checklist for Diagnostic and Statistical Manual of Mental Disorders-Fifth Edition (PCL-5) to measure PTSD symptoms [52]. The PCL-5 consists of a list of 20 problems (α = .93) and requires participants to rank how much they have been bothered by these in the past month on a five-point Likert scale ranging from 0 (‘not at all’) to 4 (‘extremely’). The established cutoff for provisional PTSD diagnosis established with PCL-5 is 33 [52].

Depression/anxiety: The Hopkins Symptoms Checklist-25 (HSCL-25) was used. This tool comprises symptoms of strain that people sometimes have, whereby 10 items focus on anxiety (α = .92), and 15 items on depression (α = .92) as defined in the Diagnostic and Statistical Manual of Mental Disorders (DSM-IV) [53]. Participants rate how much each symptom has bothered them in the last month on a four-point Likert scale ranging from 1 (‘not at all’) to 4 (‘extremely’). Mean scores of 1.75 and higher on the HSCL-25 are commonly recognized as predictive of clinical risk for depression and anxiety [54].

Hazardous alcohol consumption: This was measured with the Alcohol Use Disorder Identification Test for Consumption (Audit-C) [55]. This brief three-item alcohol screen focuses on the frequency and quantity of alcohol consumption (α = .64). Audit-C test scores of 3 or more for women and 4 or more for men are considered positive, indicating a heightened risk for hazardous drinking/alcohol use disorder [56].

Burnout: We adopted the Maslach Burnout Index Human Services Survey [57]. Through a seven-point Likert scale, participants indicate how often they have job-related feelings related to emotional exhaustion (EE) (α = .85) and depersonalization (DP) (α = .83). Established cutoffs for high levels of burnout are 27 points and above on the EE subscale, and 13 points and above on the DP subscale [57]. We excluded from analysis the responses for the sub-scale of personal accomplishment (PA), due to its marginal internal consistency as shown by Cronbach’s alpha (α = .67) and due to its marginal relevance to the burnout syndrome [58].

#### Predictor variables

We collected data on socio-demographic characteristics and nine main constructs based on their relevance in previous research on the mental health of humanitarian workers [17–20,22,24,26]. These are stress levels and trauma exposure (job demands); perceived organizational work experience, organizational support, team cohesion (job resources); and perceived social support, spiritual transcendence, coping, and health habits (personal resources).

Stress Levels: These were measured through a list of 18 chronic stressors. Based on Ager et al. [17] and adjusted to cover stressors specific to the South Sudan context, these include ‘uncertainty about political stability’, ‘traffic difficulties’, ‘excessive heat’, and ‘separation from close relatives’. Participants reported if they are currently experiencing these stressors, and the levels of stress each item currently causes them on a scale ranging from 0 (‘no stress’) to 4 (‘extreme stress’). A continuous variable was computed to reflect participants’ overall exposure to chronic stress.

Trauma Exposure: We assessed this through a list of 25 traumatic events based on the Harvard Trauma Questionnaire [59] and adjusted by covering events specific to the South Sudan context. This list included events such as ‘being expelled from the country’, ‘being shot at’, ‘serious road/vehicle accident’, and ‘unexpected or premature death of family member or colleague’. Participants indicated if they have witnessed, experienced, or not been exposed to these events in the past 10 years. The number of traumatic events witnessed and experienced was summed up to a composite score of trauma exposure ranging from 0 to 25.

Organizational Work Experience: Taken from previous work by Ager et al. [17], four six-point scale items ranging from 1 (‘strongly disagree’) to 6 (‘strongly agree’) were used to assess participants’ perceptions of work within their organization. Examples are ‘my organization encourages me to take vacation and sick leave’, and ‘my organization helps to manage team conflicts effectively’ (α = .72).

Organizational Support: We used two sets of questions from Ager et al. [17] and adjusted these to match the South Sudan context: The first set asked about the provision of 14 support items and benefits, such as communication equipment and health insurance. The second set asked about the provision of briefings and trainings on 11 topics, including stress management and security risks and protocols. Two composite scores were calculated reflecting the total sum of items and benefits, and trainings and briefings received.

Team Cohesion: Two scales were established to assess relationships among co-workers and with management based on four and six 5-point Likert scale questions, respectively [60]. While the items on team cohesion among co-workers (α = .84) focused on aspects such as spending time together outside office hours and considering colleagues friends, team cohesion with management (α = .86) addressed topics such as micromanagement and interest in personal welfare. Scores on these scales ranged from 5–20 and 5–30.

Social Support: The selection of 12 4-point Likert scale questions extracted from the Social Provisions Scale used by Ager et al. [17] was adopted. Ranging from 1 (‘strongly disagree’) to 4 (‘strongly agree’), a composite score was computed reflecting participants’ perceived social support (α = .80).

Spiritual Transcendence: We took four questions from the Spiritual Transcendence Index developed by Seidlitz et al. [61] to measure this concept. Participants indicated whether they are religious and/or spiritual and their level of agreement with statements such as ‘my spirituality gives me a feeling of fulfillment’, and ‘I experience a deep communication with God’, with 1 indicating ‘strong disagreement’ and 6 ‘strong agreement’ (α = .96).

Coping: The Brief COPE was used to assess coping strategies as suggested by Cooper et al. [62]. These are emotion-focused strategies (α = .78), problem-focused strategies (α = .81), and dysfunctional strategies (α = .80). The Brief COPE has been validated in many cross-cultural settings (e.g., [63,64]).

Health Habits: A health habits index was created based on previous work by Eriksson et al. [19] and adjusted to the study at hand (i.e., items accounted for by other measures were excluded from this index to avoid duplication). This index summed positive responses for the following four items: consuming 10 or more servings of fruits, vegetables or their juices per week; less than seven servings of junk food per week; less than two servings of caffeinated beverages per day; and engaging in physical exercise three times or more per week. The score for this index ranged from 0–4.

### Data analysis

To select predictors with the highest predictive power, we applied Least Absolute Shrinkage and Selection Operator (LASSO) regression. LASSO is a type of machine learning that performs more stable and accurate variable selection than other commonly used methods, such as best subset selection, forward selection, backward elimination, and stepwise regression; it is the superior alternative [65–67]. LASSO is especially suitable for large p small n datasets [68] and selects fewer noise predictors than commonly used variable selection methods [69]. Given its unique features, LASSO is increasingly applied in mental health research, for instance on PTSD [70] and substance use [71].

As required for LASSO, we established six sub-sets of data, each including complete cases of one mental health outcome and all predictor variables. We randomly split these into training and test sets (50:50). Models for each mental health outcome were trained on the respective training set. To assess performance regarding prediction accuracy, we calculated mean squared errors (MSEs) for these models on the test sets. The MSEs of all sparse models established through LASSO were smaller than those of the models including all predictor variables. This confirms their better performance regarding prediction accuracy. To obtain final coefficients and perform inference we then ran linear regression analyses (‘OLS post LASSO’) on each data sub-set with the predictors selected through LASSO [72,73]. Regression analyses were undertaken with the package “glmnet” in R 1.0.143.

## Results

### Survey participation

Forty-five organizations out of the 124 that were contacted confirmed their willingness to support of the survey. This included 21 national NGOs (NNGOs), 20 international NGOs (INGOs) and 4 UN entities, all of which fulfilled the eligibility criteria. One organization clarified being currently inactive in South Sudan, one anticipated being denied funding through the HRP, four declined the request, and 73 did not respond.

Based on information received from supporting organizations, the number of humanitarian workers who had access to the survey was estimated to be between 2672 and 3238. A total of 277 humanitarian workers completed the survey (a response rate in the order of 10%). This rate is in line with comparable research undertaken through online surveys in conflict-affected states (e.g., [74]) and the estimated average response rate for external online surveys [75].

About three quarters of survey participants were male (78%). Approaching half (48%) of participants were aged between 30–39 and 40% had a Bachelor degree as their highest level of education. A slight majority of participants were based in Juba (54%), classified themselves as national staff (52%), and were employed by INGOs (64%). Thirty-five per cent of participants worked as Managers/Coordinators, the largest job category for those completing the survey. These and further socio-demographic characteristics are presented in Table 1.

**Table 1:** Socio-demographic characteristics of the cohort.

Variable	N	%
*Duty station*		
Capital (Juba)	149	53.8
Field	128	46.2
*Gender*		
Female	61	22.0
Male	216	78.0
*Age*		
<30	51	18.4
30–39	134	48.4
40–49	67	24.2
50+	25	8.1
*Civil status*		
Single/separated/divorced/widowed	52	18.8
In a committed relationship/married	223	80.5
*Level of education*		
Secondary school / High school	19	6.9
Higher vocational education/technical training	41	14.8
University (BA)	112	40.4
Postgraduate (MA, MSc, PhD)	105	37.9
*Type of organization*		
National NGO	58	20.9
International NGO	176	63.5
UN entity	41	14.8
Other	2	0.7
*Contract type*		
National staff	144	52.0
International staff	119	43.0
Consultant	9	3.3
UN Volunteer	5	1.8
*Job function*		
Country Director / Head of Mission	18	6.5
Manager/Coordinator (Programme or Operations)	96	34.7
Technical/Programme	63	22.7
Logistics	12	4.3
Administrative	21	7.6
Human Resources	15	4.3
Other	52	18.8
*Working directly with beneficiaries*		
No	93	33.6
Yes	184	66.4
*Previous humanitarian field assignments*		
10 or more	25	9.0
5–9	49	17.7
2–4	130	46.9
1	46	9.7
None	27	9.7
*Years spent in South Sudan as humanitarian worker*		
<1	39	14.1
1–2	41	14.8
2–3	42	15.2
3–4	27	9.7
4–5	23	8.3
5–6	23	8.3
>6	82	29.6
*Mental health history (received counseling or medication for emotional problem)*		
No	225	81.2
Yes	46	16.6

Note: Effective n ranges from 271 to 277.

### Chronic stress exposure

Participants were exposed to chronic stress; the average number of chronic stressors experienced was 16.31 (SD = 3.47), and the overall mean score on the stress level scale was 29.37 (SD = 14.04). The three stressors most frequently identified by participants as causing extreme stress related to the uncertainty about the political stability in the country (41%); travel difficulties, restrictions on movements, threatening checkpoints, and rough roads (38%); and the separation from close relatives due to work responsibilities (23%) (Table 2).

**Table 2:** Percentage of participants reporting extreme stress.

Stressors	%
Uncertainty about the political stability	40.9
Travel difficulties, restrictions on movements, threatening checkpoints, rough roads	37.9
Separation from close relatives due to work responsibilities	22.9
Armed security, needing to have armed guards at work or living place	18.8
Feeling powerless to change the situation of the beneficiary community	16.5
Excessive heat, cold, or noise	15.9
Economic/financial problem	15.5
Workload expected by organization is too high	14.5
Feeling powerless to change one’s own situation	13.9
Feeling hostility from others due to one’s affiliation to a certain group (e.g. tribe, nationality, religion)	13.8
Lack of recognition from organization management for work accomplishment	13.1
Tension due to expatriate and national staff not treated equally by organization management	11.6
Being asked to perform duties that are outside of one’s professional training or terms of reference	11.3
Lack of recognition from the beneficiary community for work accomplished	8.8
Conflicts or misunderstandings between co-workers	7.9
Criticism of work by media or beneficiary community members	7.2
Lack of direction from organizational management	6.5
Criticism of work by organization management	5.1

Effective n ranges from 273–277.

### Coping strategies

Cooper et al. [62] established three coping strategies: emotion focused coping, involving the use of emotional support, positive reframing, acceptance, religion, and humor; problem focused coping, involving active coping, planning, and use of instrumental support; and dysfunctional coping, involving venting, denial, substance use, behavioral disengagement, self-distraction, and self-blame. Table 3 shows coping strategy mean scores of survey participants.

**Table 3:** Coping strategy.

Coping strategy		M	SD	Range
Emotion focused		25.23	5.99	10–40
Problem focused		17.39	4.20	6–24
Dysfunctional		23.19	6.35	12–48

M = mean; SD = standard deviation. Effective n ranges from 253–261.

### Mental health outcomes

Applying the established cutoff for provisional PTSD diagnosis, 24% of all survey participants experienced symptoms indicative of high risk of post-traumatic stress. Disaggregated by contract type, 36% of national and 13% of international staff met symptom thresholds associated with a diagnosis of PTSD; this difference was significant [χ^2^ (1, N = 231) = 16.51, p < .001].

Just over one-third (39%) of survey participants scored at or above the established cutoff suggestive of depression, and 38% scored at or above the cutoff suggestive of anxiety disorder. We found a higher proportion of national staff meeting symptom thresholds associated with disorder than international staff: for the former 45% and 52% reached scores suggestive of depression and anxiety disorder, respectively; for the latter 35% and 24% reached these thresholds. While no significant association was found between contract type (national/international staff) and provisional depression diagnosis [χ^2^ (1, N = 244) = 2.62, p = .11], the association between contract type (national/international staff) and anxiety disorder was significant [χ^2^ (1, N = 247) = 20.65, p < .001].

A third of both female (36%) and male (35%) participants reached positive Audit-C test scores. Prevalence of reported hazardous drinking/alcohol use disorder was significantly higher amongst male international staff than male national staff [χ^2^ (1, N = 205) = 13.68, p < .001]: Twenty-five percent of male national staff and 50% of male international staff reached Audit-C thresholds. Amongst women there was a similar trend, with 21% of female national staff and 41% of female international staff reporting drinking at levels suggestive of disorder. However, this difference was not significant [χ^2^ (1, N = 58) = 2.27, p = .13].

Based on the established cutoffs, 24% of all survey participants fulfilled the criteria for high burnout on the EE sub-scale, and 19% on the DP sub-scale. International staff reported higher rates of difficulty than national staff: 29% and 23% of international staff reached scores suggestive of burnout case on the EE and DP sub-scales respectively compared with 19% and 18% of national staff on these same sub-scales. The associations between contract type (national/international staff) and EE [χ^2^ (1, N = 237) = 3.72, p = .05] and contract type (national/international staff) and DP [χ^2^ (1, N = 250) = 1.03, p = .31] were not significant.

### Regression analyses

Higher levels of chronic stress (p < .001) and trauma exposure (p < .01) were risk factors for PTSD, with those participants experiencing higher levels of stress and trauma being significantly more likely to report PTSD symptoms.

Dysfunctional coping was significantly positively associated with depression (p < .001). Further, participants experiencing higher levels of chronic stress were significantly more likely to experience symptoms of depression than those who felt less stressed (p < .001).

The risk for anxiety increased with being a woman (p < .05). The number of years spent in South Sudan as humanitarian worker was significantly positively associated with anxiety symptomatology (p < .05). Anxiety was also significantly positively related with dysfunctional coping (p < .01) and exposure to stress (p < .001).

Hazardous alcohol consumption was significantly associated with duty station (p < .01); humanitarian workers based in the capital were at greater risk of engaging in hazardous drinking than those based in the field. Spiritual transcendence was negatively related to hazardous alcohol consumption (p < .001) (Table 4).

**Table 4:** Linear regression models for PTSD, depression, anxiety, and hazardous alcohol consumption.

Outcome	PTSD (n = 105)		Depression (n = 108)		Anxiety (n = 109)		Hazardous alcohol consumption (n = 111)	
Predictors	B	SE	B	SE	B	SE	B	SE
Gender					-0.16*	0.08	0.15	0.08
Duty station							-0.22*	0.08
Years in South Sudan					0.18*	0.08		
Mental health history					-0.02	0.08	0.14	0.08
Spiritual transcendence							-0.32***	0.09
Perceived social support					-0.15	0.09	0.02	0.09
Organizational support–benefits/ items							0.21	0.09
Team cohesion—management							0.01	0.09
Organizational work experience					0.08	0.08		
Coping–problem focused					0.06	0.09		
Coping–dysfunctional			0.37***	0.07	0.28**	0.09	0.14	0.08
Chronic stress	0.48***	0.08	0.52***	0.07	0.34***	0.08		
Trauma exposure	0.29***	0.08			0.16	0.09		

PTSD = Posttraumatic Stress Disorder; Years in South Sudan = Years spent in South Sudan as humanitarian worker; PTSD adjusted R^2^ = .39; Depression adjusted R^2^ = .46; Anxiety adjusted R^2^ = .36; Hazardous alcohol consumption adjusted R^2^ = .31

*p < .05

**p < .01

***p< .001

B = standardized beta; An additional 12 predictors were included in LASSO regressions but had coefficients of zero for all outcomes. These predictors are age, education, civil status, contract type, organization type, job function, number of previous humanitarian field assignments, working directly with beneficiaries, coping–emotion focused, organizational support–trainings/briefings, team cohesion–co-workers, and health habits.

In terms of burnout (Table 5), working directly with beneficiaries was associated with lower levels of EE (p < .01), while greater risk of EE was associated with higher levels of chronic stress (p < .01). Higher scores on DP were associated with chronic stress (p < .001). While higher levels of team cohesion with co-workers were associated with lower levels of DP (p < .05), higher levels of team cohesion with management were associated with higher levels of DP (p < .05).

**Table 5:** Linear regression models for burnout.

Outcome	Emotional exhaustion (n = 104)		Depersonalization (n = 108)	
Predictors	B	SE	B	SE
Education	0.06	0.09	-0.15	0.10
Civil status	-0.05	0.09		
Contract type			0.14	0.09
Organization type	-0.16	0.09		
Duty station			0.03	0.10
Working directly with beneficiaries	-0.26**	0.09	-0.15	0.10
Mental health history			0.04	0.09
Spiritual transcendence	-0.18	0.10		
Perceived social support	-0.08	0.09		
Organizational support–trainings/ briefings	-0.15	0.09		
Organizational work experience	-0.07	0.10		
Team cohesion–co-workers			-0.21*	0.09
Team cohesion–management			0.20*	0.09
Coping–dysfunctional	0.11	0.09		
Chronic stress	0.30**	0.09	0.39***	0.10
Trauma exposure			0.18	0.10

EE adjusted R^2^ = .27; DP adjusted R^2^ = .20

*p < .05

**p < .01

***p< .001

B = standardized beta; An additional nine predictors were included in LASSO regressions but had coefficients of zero for all outcomes. These predictors are gender, age, job function, number of previous humanitarian field assignments, years spent in South Sudan as humanitarian worker, organizational support–benefits/ items, coping–problem focused, coping–emotion focused, and health habits.

## Discussion

### Symptom burden of mental health outcomes

Our results suggest that humanitarian workers in South Sudan experience substantial levels of symptom burden of PTSD, depression, anxiety, hazardous alcohol consumption and burnout. Differences in the prevalence rates established for national and international staff were significant only for PTSD, anxiety, and hazardous alcohol consumption among male staff.

It is challenging to identify reference groups for meaningful comparison of the established prevalence rates. Further, comparing health measures across groups within a society and across different societies is–as well-known–problematic [76]. Nonetheless, the prevalence of symptoms indicative of high risk of post-traumatic stress we found among national staff (36%) is higher than the rates for mild or moderate forms of PTSD among South Sudanese refugees in northern Uganda (15–20%) [77]. It is lower than the prevalence consistent with PTSD diagnosis found among civilians from multiple locations across South Sudan (41%) [78]. The prevalence we found (36%) is also lower than that found among respondents based in Malakal Protection of Civilians side (53%) [78]. The proportion of South Sudanese national staff reaching scores above thresholds for anxiety (52%) is as high as that of Ugandan national staff operating in neighboring Gulu assessed with the same tool (53%) [17].

Regarding international staff, the prevalence rates we found for PTSD and anxiety generally exceed those of western adult populations [79–81]. Likewise, they exceed the proportion of expatriate humanitarian workers in Kosovo who scored above cutoffs for PTSD (13% and 1% respectively) and anxiety (24% and 9% respectively) [20]. The anxiety prevalence we found among international staff based in South Sudan also exceeds the rate established by Cardozo et al. [26] for post-deployment anxiety (12%) among humanitarian workers across countries.

Few studies looked at alcohol consumption. The recent study by Jachens, Houdmont and Thomas [23] investigated alcohol consumption among humanitarian workers, including national and international staff. Using the same measure that we used, this study found that 18% of all female and 10% of all male participants engaged in hazardous drinking [23]. Lopes Cardozo et al. [20] report prevalence of hazardous alcohol consumption for national and international staff (3% and 16% respectively) but neglect disaggregation by gender. The prevalence rates we found among female humanitarian workers in South Sudan and male national and international staff (36%, 25% and 50% respectively) all exceed the rates established by these studies.

### Trauma and chronic stress exposure

Although experience of traumatic events was associated with higher reportage of symptoms of PTSD, it was chronic stress that was most consistently associated with mental health problems. This confirms our hypothesis from consideration of the JDR model that job demands are significantly positively associated with mental health problems. Our finding is in line with results from other research on the impact of stress versus major life events in adult population samples [82]. It also reinforces the results of previous work that emphasizes the cumulative effect of daily stress in contexts of conflict and instability [12,17,19]. Previous research on humanitarian workers’ mental health has a strong concentration on trauma exposure (and PTSD as the corresponding “signature” disorder) (e.g., [20,21,83]). Future studies should consider integrating an increased focus on the effects of chronic stress.

Exposure to traumatic events and especially chronic stress is inevitably a characteristic of the operating environments of humanitarian workers. Nevertheless, there is a clear case for preventive measures addressing security and work conditions on the basis of our finding. With regards to South Sudan, developing and disseminating thorough security and evacuation protocols and re-confirming with staff that they are covered in case of need is one remedy that may help mitigate top-ranked stressors such as uncertainty about the political stability. Investments in comprehensive briefings prior to deployment on the implications of humanitarian work on staff’s personal life, and in staff support services that promote work-life balance (e.g., rest and recuperation) may help address stress caused by family separation and high workloads.

### Coping and spiritual transcendence

Surprisingly, we found no significant association between emotion focused and problem focused coping strategies and mental illness. This is contrary to expectations from studies with other population groups using the same measure that found significant relationships between one or both coping strategies and depression or anxiety [84,85]. The positive relationship we found between dysfunctional coping and depression and anxiety is in line with these studies [62,86]. Specifically with expatriate humanitarian workers, Eriksson et al. found a significant positive association between avoidant coping and PTSD, anxiety, and depression. Lopez Cardozo et al. [22] found using an avoidant coping style significantly associated with more anxiety among national humanitarian workers. These findings suggest potential for addressing coping strategies adopted by humanitarian workers. However, establishing appropriate, protective forms of coping in humanitarian settings may be challenging. Coping strategies in situations of conflict may, as suggested by recent work in conflict-affected populations [87], require distinct adaptation to be effective.

We found the concept of spiritual transcendence [88] to be negatively associated with hazardous alcohol consumption, indicating that humanitarian workers with higher spirituality are less likely to engage in such harmful behavior. This finding matches outcomes from similar occupation groups [89]. Our hypothesis that personal resources are significantly negatively associated with mental health problems holds for this construct.

### Team cohesion and working with beneficiaries

The association of higher levels of team cohesion with co-workers with lower levels of depersonalization reinforces the value of establishing effective team working in high-stress environment suggested in other work (e.g., [17]). However, it was unexpected that higher team cohesion with management was associated with higher rather than lower levels of depersonalization. A possible explanation is that in the humanitarian worker sample at hand, depersonalization was most prevalent among Managers/Coordinators. It is well known that burnout has a spillover effect and aspects associated with depersonalization, such as pessimistic attitudes towards beneficiaries, can spread from managers to team members [90]. Our hypothesis derived from the JDR model that job resources are negatively associated with mental health problems is thus partially fulfilled.

### Socio-demographic factors

The observation that working directly with humanitarian aid beneficiaries was associated with lower not higher levels of emotional exhaustion points to the stresses in this environment associated with managerial and coordination functions, too. The fact that duty station was associated with hazardous alcohol consumption can most readily be attributed to the fact that Juba–the capital–provided far easier physical and social access to alcoholic beverages (e.g., [91]). On the basis of studies in other humanitarian settings (e.g., [17,19,20,92]), we had anticipated further socio-demographic characteristics to explain variance in the mental health outcomes. However, while this study found some of the differences in the established prevalence rates for national and international staff to be significant during bivariate analyses, contract type was not a significant predictor during multiple regression analyses. Risk factors for common mental health problems specific to gender, especially gender-based violence and sexual harassment, are widespread within humanitarian communities (e.g., [93,94]), yet gender was only significantly associated with anxiety. In light of this and previous studies with humanitarian workers that have been inconstant in their findings regarding gender differences (e.g., [17]), we are especially interested in rates of reported mental ill-health between men and women. In the absence of such findings, the factors that influence the relationship between gender and mental health among humanitarian workers remains unclear [16].

### Limitations

This study has four main limitations. Firstly, the cross-sectional survey design does not provide a basis for establishing cause-effect relationships. Secondly, we could not conduct clinical interviews to establish psychiatric diagnosis. Instead, we used screening tools that have exhibited strong psychometric properties in many settings and have been shown to be suggestive of rates of clinical disorder (e.g., [48–51]). Thirdly, our sample may not be fully representative of the humanitarian community in South Sudan. Although data on the composition of the humanitarian community in South Sudan are scarce, recent estimates suggest that national staff make up 92% of the NGO staffing [95], in line with the global estimate of 90% of the humanitarian workforce in field locations [40]. By contrast, our sample comprises of 52% national and 43% international staff. However, our sample reflected the humanitarian community well in other regards, such as the greater proportion of male humanitarian workers and level of education: men made up the majority of the sample and most had a University degree, in line with related studies on humanitarian workers (e.g., [20,21,24]). Lastly, the low survey response rate clearly raises cautions regarding the generalizability of the study results, and further research is required to confirm our findings. Organizations repeatedly identified limited Internet access as a hurdle, especially for field-based staff. The English language in which the survey was conducted may have presented an additional barrier for participation for some staff. However, the response rate is well in line with response rates typically received by online surveys [75,96].

## Conclusion

Our results suggest that humanitarian workers in South Sudan experience substantial rates of mental health problems. This study highlights that chronic stress plays a dominant role in understanding mental health problems. It points to the need for organizational staff support strategies that mitigate humanitarian workers’ chronic stress exposure. Dysfunctional coping was associated with higher risk for mental disorder, indicating an important role for education of humanitarian workers regarding stress management. However, strategies that may be adaptive in other contexts may not be effective in high stress environments such as that studied here.
